# Medical expert reports in occupational health: a bibliographic review

**DOI:** 10.47626/1679-4435-2020-544

**Published:** 2020-12-11

**Authors:** Rômulo Garcia Mazanti, Thainan Alves Silva, Stela Almeida Aragão, Raí Novaes Nogueira, Roberto Del Valhe Abi-Rached

**Affiliations:** 1 Especialização em Perícias Médicas, Faculdade Unyleia - Jequié (BA), Brazil; 2 Mestrado em Ciências da Saúde, Universidade Estadual do Sudoeste da Bahia (Uesb) - Jequié (BA), Brazil; 3 Medicina, Universidade Estadual do Sudoeste da Bahia (Uesb) - Jequié (BA), Brazil; 4 Doutorado em Medicina, Universidade de São Paulo - São Paulo (SP), Brazil

**Keywords:** expert testimony, occupational health, forensic medicine, formal work, medical examination

## Abstract

Forensic medicine is a branch of medicine located within both the health and law domains that contributes in issues that include civil, criminal, and labor law. Within labor law, medical expert reports aim to evaluate the capacity of workers and ensure them their rights and duties. This study aimed to discuss the information available on the scientific literature regarding expert reports in occupational health. This is a bibliographic review ranging the period of 2012 to 2018 that searched the MEDLINE, PubMed, and LILACS databases, as well as official documents on the subject. We selected 7 articles corresponding to the initially proposed criteria and read them thoroughly. This allowed us to establish 3 main themes corresponding to the study results: 1) The importance of expert reports in occupational health; 2) Hurdles in the context of expert reports in occupational health; and 3) The role of the medical expert in labor lawsuits. Through the selected studies and the evaluation of the initial proposition of this review, we confirmed the importance of expert reports in ensuring workers’ rights in occupational health. Moreover, this study allowed the identification of gaps in studies regarding the role of expert reports in occupational health, thus more research should be encouraged in view of the great social relevance of this theme.

## INTRODUCTION

Forensic medicine is a multidisciplinary science, that is, it can be classified and used both in the medicine and law domains; it includes areas such as forensic anthropology, medicolegal practice, forensic toxicology, and genetics. The main objectives of this domain of medicine include providing legal services and aiding in investigations. It is worth noting that the activities performed by forensic medicine professionals and medical expert reports are applied beyond criminal law, also contributing to civil, occupational, social security, insurance, and administrative issues, as well as medical audits.^1^

According to the Civil Procedure Code of 2015, when considering evidence that relies on technical or scientific knowledge, the designated judge should be advised by an expert, known as expert witness. In the area of forensic medicine, a qualified physician should be chosen by the judge and is named a medical expert witness.^2^ The medical expert witness can act in judicial and non-judicial procedures; the first involves social security, civil, labor, and criminal law and the second includes expert reports required by institutions such as the National Social Security Institute (INSS), as well as audits and reports required by insurance companies.^3^

The Portuguese word for expert report (“*perícia*”) comes from the Latin word “*peritia*,” meaning special ability, and highlights that this professional should be graduated and have specialized scientific and technical knowledge. Therefore, the main occupation of an official health expert is to evaluate the capacity of a worker in view of a disease or injury, an occupation that requires technical skills and abilities, as well as knowledge on occupational health, epidemiology, and civility.^4^ Within the area of labor law, occupational activities are known to have negative consequences in the conditions and quality of life of workers, especially considering their health.^5^ On the other hand, work is intimately associated to one’s personal identity and provides purpose in life, thus ensuring a right of citizenship, to achieve one’s aspirations and projects, and ultimately provides satisfaction.^6^

Considering these aspects, understanding that health care is a human right that should be provided by the state through social and economic policies is of utmost importance. These policies should aim to reduce risks of diseases and other disorders and provide universal and equal access to actions and services that promote, protect, and restore health.^7^ However, in Brazil, 700 000 occupational accidents go unreported each year, in addition to events not officially reported by INSS. Occupational accidents consider commuting accidents, typical occupational accidents, as well as work-related diseases and disorders.^8^

In 2012, directive No. 1823 instituted the National Policy on Occupational Health (PNSTT) in order to structure occupational health actions, with the main objective of defining principles, guidelines, and strategies to be adopted by the 3 spheres of government that manage the Unified Health Service (SUS): To provide full attention to workers’ health, emphasizing surveillance, in order to promote and protect workers’ health and reduce morbidity and mortality caused by production processes.^9^ Health care services should consider the inclusion of workers in the labor process, taking into consideration the fact that work is a decisive aspect for the health-disease process. Health care teams should be aware of the occupational activities performed by the patients/workers in order to develop actions, within the SUS care network, that promote, protect, assist, and rehabilitate these workers.^10^

In this context, a medical expert report is not only a medical procedure based on clinical data, but also an essential tool for evaluating workers’ health, since it evaluates the multifactorial causality of sickness leaves and not only the signs and symptoms presented by an individual. Therefore, multi-professional reports contribute to the foundation and definition of a conclusive expert report.^4^

The work/health relationship is complex and requires different actions from public institutions that, in Brazil, consist of different ministries, which should act to provide intersectoral and comprehensive care.^5^ Studies that focus on forensic medicine, mainly those considering occupational health, allow a necessary verification of the performance of medical expert reports, which are fundamental elements for amplifying the common biomedical perception that considers only the disease, without regarding the whole individual. This highlights the need for evaluating causal aspects of the onset and maintenance of diseases and the importance of expert reports as a means of promoting health and reducing damages in accordance with social, cultural, environmental, occupational, economic, and political diversities.

Finally, we have established the following research question: What are the uses of expert medical reports in occupational health? From this question, we elaborated our objective of discussing the information available on the scientific literature regarding this subject.

## METHODS

We performed a bibliographic review of the literature through a retrospective search for scientific articles on medical expert reports in occupational health, within the domain of forensic medicine. The literature review is essential in scientific investigation and involves the search, analysis, synthesis, and interpretation of information related to the studied subject found in sources such as scientific journals, books, and abstracts. This mechanism of grouping and analyzing data is essential for defining the problem and observing the current state of knowledge and its gaps, in addition to the contribution of the investigation for the subject in question.^11^

Our search was performed in October and November 2018 with the Portuguese and English descriptors for “expert testimony,” “occupational health,” and “forensic medicine.” We used the Boolean operator “AND” between the primary descriptor “expert testimony” and the other 2 secondary descriptors in a search for titles, abstracts, and keywords in each of the following databases: MEDLINE, PubMed, and LILACS.

The selected articles should agree to the following criteria: full-text versions available online; “expert testimony,” and “occupational health” or “expert testimony” and “forensic medicine” as main subjects; studies performed with human participants; studies published in English and/or Portuguese between 2012 and 2018. Our exclusion criteria considered incomplete studies, those that did not answer our guiding question, and duplicate articles.

**Figure 1:**
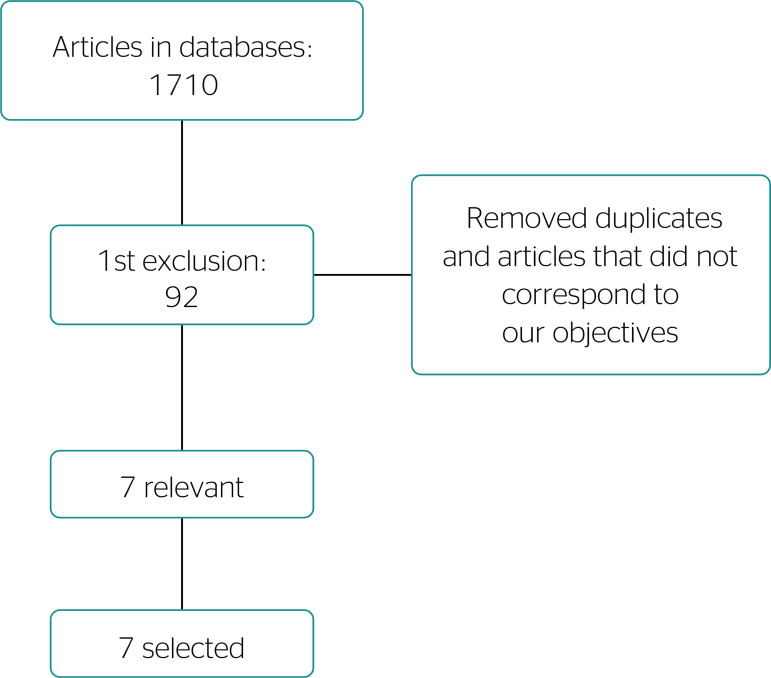
Article selection flowchart. Jequié, state of Bahia, 2018 (N = 7).

Considering all databases, we initially found 1710 articles. After adopting the inclusion and exclusion criteria and reading the article titles, we selected 92 articles for a first glance at the abstracts. Articles that did not correspond to our subject were excluded, and finally we selected 7 articles (Table 1) in accordance with our criteria. These articles were fully read and compared in order to emphasize their aims and methods, as well as differences and idiosyncrasies between studies, finally contextualizing them in this review (Figure 1). Moreover, we reviewed other sources and official documents considering medical expert reports in occupational health and labor law associated to forensic medicine.

**Table 1:** Selected articles, authors, study designs, titles, subjects, and years of publication. Jequié, state of Bahia, 2018 (N = 7).

Author	Study design	Title	Subject	Year
Melo^12^	Qualitative	Governo da população: relação médico-paciente na perícia médica da previdência social	Forensic medicine and medical expert reports	2014
Lise^13^	Bibliographic search	Isenção e autonomia na perícia médica previdenciária no Brasil	Medical expert reports	2013
Melo^14^	Qualitative	Moralidade e risco na interface médico-paciente na perícia médica da Previdência Social	Medical expert reports and forensic medicine	2014
Siqueira^15^	Qualitative	As LER/DORT no contexto do encontro simbólico entre pacientes e médicos peritos do INSS/SP	Medical expert reports and occupational health	2013
Cantley^16^	Epidemiologic	Expert ratings of job demand and job control as predictors of injury and musculoskeletal disorder risk in a manufacturing cohort	Medical expert reports and occupational health	2016
Pinto Júnior^17^	Exploratory	Evolução da saúde do trabalhador na perícia médica previdenciária no Brasil	Medical expert reports and occupational health	2012
Chaves^18^	Bibliographic search	Judicialização da medicina e o impacto orçamentário na administração pública: uma abordagem médico-legal	Forensic medicine and medical expert reports	2017

## RESULTS AND DISCUSSION

After reading the selected material, we established 3 main themes for our results: (1) The importance of medical expert reports in occupational health; (2) Hurdles in the context of expert reports in occupational health; and (3) The role of the medical expert in labor lawsuits.

### THE IMPORTANCE OF MEDICAL EXPERT REPORTS IN OCCUPATIONAL HEALTH

Work-related medical expert reports are required for obtaining social security benefits provided by INSS, thus representing an important pillar for the protection of workers that are unable to obtain their means of living through work. The elaboration of such reports requires wide knowledge both within the medicine and law fields.^13^ In forensic medicine, there are no financial incentives regarding the amount of favorable or unfavorable reports. The right to a sickness benefit is currently ensured to those that fit the requisites characterizing them as ensured and incapable, that is, this benefit is destined for workers whose diseases cross their physical and psychological states to the point that they become incapacitated for their occupational activities.^12^ Medical expert assessments required in occupational health settings thus aim to analyze the consequences of a harm to the work capacity of an individual instead of diagnosing a disease.^13^

In this context, experts consider the physical examination to be an extremely necessary evaluation for elaborating and concluding expert reports, since other examinations such as imaging and laboratory tests are considered to be of secondary importance.^15^ Therefore, medical expert reports are needed in the evaluation of workers’ health as an essential medicolegal tool, produced by an expert, that describes the work capacities of an individual.^13^ In addition, a study on injury risks and musculoskeletal disorders in a cohort of manufacturing industry workers highlighted the importance of identifying, monitoring, and reducing work-related diseases and disorders in the promotion of health and safety. This study confirms the importance of medical expert reports in occupational health.^16^

### HURDLES IN THE CONTEXT OF EXPERT REPORTS IN OCCUPATIONAL HEALTH

Within the occupational health field, medical actions do not aim to restore health, provide treatment, or prevent a disease or disorder. In this context, there is no commitment to assistance, since the medical expert activity involves steps such as anamnesis, physical examination, and the reading and interpreting of medical reports and complementary examinations.^14^

Physicians are pressured to be held responsible for expert reports, their interpretation and examination, or for the refusal of legal and grounded facts. Therefore, the professional activity of an occupational physician should aim to, even in the face of uncertainty, analyze the causal and disability circumstances according to the legal order and its bureaucratic implications, thus becoming a difficult task.^15^ Regarding occupational health, the medical expert activity is developed among factors that compromise the expert’s autonomy, such as: (1) limitations in the elaboration of an expert medical report; (2) time-related hurdles; (3) absence of a specific area for elaborating the report; (4) a relationship of dependency with INSS; (5) lack of institutional tools. These factors directly interfere in the elaboration of a medical expert report and consequently lead to more fragile and error-prone reports.^13^

The aim of expert reports is the disease; the worker’s health is thus not involved. This model is now considered outdated, since the quantitative goals set for forensic physicians do not correspond to society’s needs because they do not consider the tendency of the disease process. This is corroborated by the apathy with which social, environmental, economic, occupational, cultural, and political issues are treated along the health-disease-incapacity process.^17^ Moreover, difficulties faced by workers in receiving diagnoses and accessing health care are related to a lack of preparation by health care professionals for performing a suitable anamnesis and answering the patients’ questions, which contribute to a delay in the beginning of treatment and in the solicitation of sickness leaves.^15^

Medical expert reports also present a conflict between the vulnerability of workers and the suitable conduct for complying with the benefit rules, since physicians face situations where there is an extreme need for the benefit, but the candidate does not fulfill the requisites for incapacity. This conflict relies on the fact that medicine traditionally seeks to alleviate vulnerability, pain, and suffering, as well as to heal; instead, forensic medicine only identifies a vulnerability and does not necessarily abide by the Hippocratic command of benevolence.^14^^,^^19^

In view of the responsibility of forensic physicians in occupational health, it is easy to observe that these are classified by workers as good physicians when they offer acceptable care and especially when they report a work incapacity, while they are considered poor physicians when they do not elaborate a favorable report for social security benefits despite proof in the form of examinations and medical records.^15^

It is important to note that the physician-patient relationship in forensic medicine is monopolized by economical and political interests of companies, unions, and the state itself.^19^ Therefore, the health policy of some companies faces problems that consist of the basic ideals of capitalism. Discussing work-related diseases raises questions about their causes. On the other hand, it is known that work-related accidents and diseases harm corporate images in the economic and public settings; this causes many work-related accidents and diseases to go unreported.^17^

### THE ROLE OF THE MEDICAL EXPERT IN LABOR LAWSUITS

Introducing workers’ health in the domain of forensic medicine, especially considering expert reports, has been an extremely important and relevant achievement that allowed the comprehension and adoption of interventions that consider aspects other than the biological issues of the health-disease process. The medical expert, in this context, is crucial for the worker to exercise his or her full rights.^20^ In a labor lawsuit, medical expert witnesses are used in cases of civil liability-moral, esthetic and material damages, as well as loss of chance-and in the return to work, both due to work-related accidents or diseases.^19^

These facts illustrate the role of medical experts and forensic medicine in issues directly related to expert reports in lawsuits and other branches of law that require medicolegal interpretations. Therefore, the absence of this type of professional would lead to concrete losses in the execution of justice, mainly resulting in judicial mistakes, since expert witnesses are currently essential in the determination of the truth.^20^

Before emphasizing the importance of the role of a medical expert witness in labor lawsuits, it is important to define some concepts of occupational health. Article 19 of Law No. 8213/1991 defines occupational accident as follows:

An occupational accident happens due to the performance of work for a company or household employer, or due to the performance of work by the insured referred to in Section 11, Subsection VII of this Law, causing bodily injury or functional impairment resulting in death, loss or reduction (permanent or temporary) of one’s work capacity.^21^ [free translation]

Section 21 of this Law considers the following cases as occupational accidents:

I. The work-related accident that, although not the only cause, contributed directly to death, reduction or loss of the work capacity of the insured, or produced an injury that requires medical care; II. The accident suffered by the insured in the work environment and during working hours, due to: a) an act of physical aggression, sabotage, or terrorism committed by a third party or a coworker; b) intentional physical offense, including by a third party, due to a work-related disagreement; c) an act of recklessness, negligence, or malpractice by a third party or a coworker; d) an act of a person under a defect of reason; e) a building collapse, flood, fire, and other unexpected events or caused by force majeure; III. The disease caused by accidental contamination during the performance of one’s occupation; IV. The accident suffered by the insured outside of the workplace and working hours: a) in the execution of an order or when performing tasks under the authority of the company; b) during spontaneous work performed for the company in order to avoid losses or to obtain gain; c) during a business trip, even if with educational purposes when financed by the company in order to better prepare its workforce, regardless of the means of transportation, including when using the insured’s own vehicle; d) in the insured’s commuting route, regardless of the means of transportation, including the insured’s own vehicle.^21^ [free translation]

Throughout the process of obtaining sickness benefits or in labor lawsuits, the work of a medical expert is of great value. This professional acts according to technical, administrative, and legal rules, evaluating the applicant (for a sickness benefit or in a labor lawsuit) and seeking a coherent conclusion about his or her health conditions and capacity of remaining at work, finally elaborating a conclusive report with medicolegal effects.^14^^,^^22^ Consequently, a medical expert should detain knowledge regarding disease pathophysiology, diagnoses and treatments, and, when focused in occupational health, consider intrinsic particularities of the worker’s occupational activities.^20^

In the medical expert report, “the forensic physician should characterize the damage suffered by the worker, the causal nexus linked to work and the circumstances that contributed to the occurrence of a work-related accident/disease, in order to assist in a legal decision.”^(23(p. 269))^ In view of this information, a medical expert has to evaluate many variables and contexts for elaborating a medical expert report that will have decisive influence in medical, social, and judicial areas.^20^ Moreover, the professional should perform a medical examination to evaluate the occurrence of an occupational accident or disease and its consequences for the worker.^23^

Within the context of medical expert reports relative to occupational health, it is necessary to distinguish the technical assistant from the medical expert. The Federal Council of Medicine (CFM), in 2000, published the characteristics of both professionals, as well as various ethical and normative guidelines that establish a comprehension of the subject and effectively regulate the role of these 2 types of medical professionals.^20^

In November 12, 2013, resolution No. 2056 of the CFM highlighted the differences between these types of physicians:

Section 52. Medical experts are subjected to the ethical principles of impartiality, respect for the individual, veracity, objectivity, and professional qualification. [...] Section 53. Technical assistants are subjected to the same principles, especially to the principle of veracity. Since they are professionals acting on behalf of one of the parties, they are not impartial. Section 54. Medical experts and technical assistants should treat each other with respect and consideration, and the expert should previously inform the technical assistants about all steps of the investigation, granting them access to all steps of the procedure.^(15,24 (p. 162-163))^ [free translation]

The work of medical professionals in the interconnections with law, especially in official expert reports, is essential for guaranteeing a fair access to health care.^18^

## CONCLUSIONS

Considering the presented studies and the initial proposition of this review, it became clear that there are gaps in the literature regarding studies that consider medical expert reports in occupational health. However, the occupational health domain, established over 3 decades ago, has been shown to have expanded the view of health sciences beyond the biomedical model and the biological phenomena towards the evaluation of the causes and reasons for disease maintenance, as well as the elaboration of health promotion and injury prevention strategies based on social, environmental, occupational, political, and economic situations.

Valuing medical expert reports and searching for alternatives in the solution of problems that hinder the suitable development of one’s occupation is a common interest of all parties involved, from physicians that chose this specialty, workers eligible for social security benefits or that seek proper health care, to the society itself, which expects improvements in public services. Therefore, the autonomy of the medical expert becomes essential in the elaboration of an expert report and in the effective care of the population. Instead, we currently have socially unprotected workers which are affected by the decline and omission of the sectors destined for their protection.

Future years will require an increasing maturity of the Judiciary regarding questions that involve the heath of the society, and mainly of its workers; people increasingly demand that their constitutional rights are materialized in their daily lives, reducing the considerable social inequality in Brazil. In view of the evidenced gaps and of the results presented in this review, we highlight the need for producing research on this topic, especially regarding the reality involving the health of Brazilian workers.
